# Effect of the extracellular component of bone marrow mesenchymal stromal cells from healthy donors on hematologic neoplasms and their angiogenesis

**DOI:** 10.1007/s13577-020-00332-y

**Published:** 2020-04-12

**Authors:** Nina Gladkova, Tomohiro Umezu, Satoshi Imanishi, Chiaki Kawana, Junko H. Ohyashiki, Kazuma Ohyashiki

**Affiliations:** 1 Kintaro Cells Power Corporation, Tokyo, Japan; 2 Department of Advanced Cellular Therapy, Tokyo Medical University, Tokyo, Japan; 3 Department of Hematology, Tokyo Medical University, 6-7-1 Nishi-shinjuku, Shinjuku, Tokyo, 160-0023 Japan; 4 Institute of Medical Science, Tokyo Medical University, Tokyo, Japan; 5 Present Address: Department of Molecular Pathology, Tokyo Medical University, Tokyo, Japan

**Keywords:** Bone marrow stromal cells, Donor age, Growth inhibitory effect, Conditioned medium, Angiogenesis

## Abstract

Bone marrow mesenchymal stromal cells (BM-MSCs) from healthy donors are a promising source of cell therapy. However, their effectiveness in cancer remains less known. This study is the first to evaluate the quality of BM-MSCs obtained from young and elderly healthy volunteers (KNT cells). The KNT cells had normal karyotypes and were positive for MSC markers (CD90, CD73, CD105). When cultured under appropriate conditions, they showed adipogenic or osteogenic potential. Hence, the anti-neoplastic effects of secretory factors [supernatant or extracellular vesicles (EV)] from KNT cells were verified using several neoplastic cells (three multiple myeloma, three myeloid leukemia, and three lymphoma cell lines). The conditioned medium (CM), but not EV, of KNT cells derived from young healthy donors significantly inhibited myeloma and lymphoma cell proliferation, but enhanced myeloid leukemia proliferation. Anti-angiogenesis effect of CM and EV derived from young KNT against hematologic neoplasia-induced angiogenesis was evident and more prominent in CM than in EV but not evident in elderly KNT-derived EV. These findings indicate that the anti-tumor effect of KNT cells depends on the types of hematologic neoplasia, with elements existing in the supernatant and not in EVs. Therefore, BM-MSC may produce soluble factors that affect cell proliferation of neoplasia, causing cell-to-cell communication. The anti-angiogenesis effect of KNT cells depends on the age of BM-MSC donors.

## Introduction

Mesenchymal stem/stromal cells (MSCs), which are found in various tissues, including the bone marrow [1, 2], umbilical cord [3], adipose tissue [4], and dental pulp [5], are a source of multipotent somatic stem cells that differentiate into bone, cartilage, and fats. Recently, MSCs have become a source of cell therapy for tissue regeneration. In addition to their multilineage potential, MSCs have gained attention because of the immunomodulatory functions of the cytokines and chemokines that they secrete. As immunomodulation by MSCs has been shown to alleviate symptoms of treatment-resistant refractory autoimmune diseases, cell transplantation therapy is increasingly being indicated for these diseases [6]: for example, treatment with bone marrow MSCs (BM-MSCs) has also been performed in acute graft versus host disease (GvHD) after hematopoietic stem cell transplantations [7]. In addition to immunoregulation by the fluid elements released by MSCs, intercellular interactions in the niches formed by cells surrounding MSCs are likely to be intricately involved in symptom alleviation [8]. Understanding these complex mechanisms is expected to lead to the development of safer and more efficient cell therapy. Recently, in addition to the above MSC-derived cytokines and chemokines, the extracellular vesicles (EVs) released by MSCs have also garnered attention [9].

Exosomes, a type of EV, are approximately 30–200 nm vesicles with a lipid bilayer membrane structure. In the late 2000s, not only proteins but also genetic materials, such as microRNA (miRNA) and mRNA, were demonstrated to be transported between cells through exosome receival [10, 11]. A recent study demonstrated the therapeutic effects of MSC-derived exosomes on GvHD, similar to MSCs [9]. However, the full scope of the biological actions of exosomes has not been well understood. A major concern with exosome use is the risk of adverse reactions. In fact, some reports revealed that exosomes secreted by abnormal MSCs can cause a cancer to be more malignant [12].

To develop safe and high-quality cell therapy with BM-MSCs, we characterized the BM-MSCs obtained from two age groups (i.e., young and elderly). Because only limited information (donor age and whether the source is from single or multiple donors) is provided on commercially available BM-MSCs, this study was performed to evaluate the quality of BM-MSCs derived from our new original source. We then assessed their growth inhibitory effect on various types of hematologic malignancies for future therapeutic application.

## Materials and methods

### Isolation of human BM-MSCs

BM-MSCs obtained from healthy 22- and 25-year-old male and 80- and 86-year-old female volunteers (young KNT cells: KNT_D_170714, KNT_L_170714; elderly KNT cells: KNT_80_170825 and KNT_86_170825, Kintaro Cells Power Co., Tokyo, Japan) were used in this study (Table 1). These cells were collected under the control of the Federal Research and Clinical Center of the Federal Medical Biological Agency of Russia (FRCC FMBA, Moscow, Russia) after approval from the institutional ethical committee (approval 5-11-03-2013 of March 03, 2013).

**Table 1:** Information of bone marrow-MSC donors

Donors	Gender	Age (years)	KNT cell ID	Doubling time (h)
UDN1	Male	22	KNT_D_170714	108
UDN2	Male	25	KNT_L_170714	108
UDN3	Female	80	KNT_80_170825	96
UDN4	Female	86	KNT_86_170825	108

### Cell culture

We seeded BM-MSCs in αMEM (Gibco, Carlsbad, CA, US) supplemented with 5% PLTMax Human Platelet Lysate (Millipore, Temecula, CA, US) and 2 U/ml Heparin (Sigma, St. Louis, MO, US) at 37 °C in a humidified atmosphere containing 5% CO_2_. Adherent cells were harvested by trypsinization and either passaged (passages 1–4) for expansion or subjected to analyses for subsequent cytogenetic analysis. The following cell lines derived from hematologic malignancies were purchased from the Health Science Research Resource Bank, JCRB Cell Bank, and ATCC: three lymphoma cell lines (SUDHL4, CRL-2957; DL40, JCRB1334; Pfeiffer, CRL-2632), three lines with multiple myeloma (RPMI8226, JCRB0034; KMS-11, JCRB1179; U266, TIB-196), and three lines with leukemia (K562, JCRB0019; HL-60, IFO50022; U937, JCRB9021). These cells were cultured in RPMI1640 (Gibco) supplemented with 10% heat-inactivated fetal bovine serum (HyClone, Logan, UT, US) at 37 °C in a humidified atmosphere containing 5% CO_2_.

### Flow cytometry and cytogenetic analysis

To characterize KNT cells using their surface antigen, the following monoclonal antibodies were used: CD90-FITC (Cat. No. 555595), CD73-APC (Cat. No. 560847), CD105-PerCPCy5.5 (Cat. No. 560819), CD34-FITC (Cat. No. 555821), CD45-PE (Cat. No. 555483), HLA-DR-PE (Cat. No. 555812), and CD271-PE (Cat. No. 557196) (BD Biosciences, San Jose, CA, US). The KNT cells were incubated with these antibodies and analyzed by an Accuri C6 cytometer (BD Biosciences), according to the manufacturer’s instructions. Chromosome analysis was performed with a conventional Giemsa-banding method after colcemid exposure. A further cytogenetic study was performed using commercially available spectral karyotyping (SKY) from SRL Inc. (Hachioji, Hino, Tokyo, Japan).

### Osteoblast and adipocyte differentiation

To confirm the adipogenic potential of young and elderly KNT cells, these cells were incubated in αMEM with 5% PLTMax until cells were confluent. Thereafter, KNT cells were cultured with adipogenic induction medium (Lonza, Basel, Switzerland). After 3 days, maintenance medium was added to cells, and three cycles of induction and maintenance media were completed. Cells were fixed with 10% formalin (Sigma-Aldrich) and stained with 0.5% Oil Red O (Sigma-Aldrich) in methanol (Sigma-Aldrich). To confirm the osteogenic potential of KNT cells, they were incubated in αMEM with 5% PLTMax until a confluent layer was achieved. Thereafter, osteogenic differentiation medium (Lonza) was added. Medium was changed every 3–4 days. After 17 days, cells were fixed in 10% formalin and stained with 10% alizarin red (Sigma-Aldrich).

### Collection of conditioned medium (CM) and EV

Young and elderly KNT cells were cultured in maintenance medium (αMEM with 5% PLTMax) which was replaced with serum-free MSCBM basal medium (Lonza). The KNT cell-derived conditioned medium (KNT cell-CM) was collected after 48 h. The collected CM was centrifuged for 15 min at 2500 rpm to remove cell and cell debris, and then concentrated (approximately tenfold concentration) using Amicon Ultra-15 3 kDa MW cut-off filter units (MilliporeSigma, Burlington, MA, US) to exclude possible contamination from lipids and metabolites. KNT cells (4 × 10^4^ cells/cm^2^) were cultured in 5 ml of MSCBM basal medium (Lonza) in a T-25 flask. The culture supernatants were harvested after 48 h of incubation, and the EV fraction was purified with Exoquick-TC reagent (System Biosciences, Palo Alto, CA, US) according to the manufacturer’s instructions. EV pellets were resuspended in 500 µl of MSCBM basal medium.

### Evaluation of the anti-neoplastic activity of various hematologic malignant cells

To evaluate the proliferative effect of KNT cells, lymphoma cells (SUDHL4, DL40, and Pfeiffer), multiple myeloma cells (RPMI8226, KMS-11, and U266), or myeloid leukemia cells (K562, HL-60, and U937) were used. Neoplastic cells (2 × 10^5^ cells/mL in maintenance medium) were cultured with or without concentrated CM or EV from KNT cells. Thirty microliters of concentrated CM or EV fractions from the KNT cells were then added to the cultured hematological malignant cell lines. Cell proliferation rates were measured using the IncuCyte Zoom live-cell analysis system (Essen Biosciences, Ann Arbor, MI, US).

### Effect on neoplastic cell-induced tube formation

Human umbilical vein endothelial cells (HUVECs) were purchased from Lonza and cultured in maintenance medium [EBM-2 medium supplemented with EGM-2 bulletKit (Lonza)]. HUVECs (300 µL of 4 × 10^5^ cells/mL in EBM-2 basal medium) were placed on a 260-µl Matrigel (BD Biosciences) in a 24-well plate and incubated overnight at 37 °C. Neoplastic cell (SUDHL4, RPMI8226, or K562)-derived concentrated CM (20 µL/well) with or without KNT cell-derived CM (20 µL/well) or KNT cell-derived EV was subsequently added. KNT cells used were KNT_D_176714 (as young KNT cells) or KNT_86_170825 (elderly KNT). After 24 h, cells were stained with calcein AM (BD Biosciences) and visualized under a fluorescence microscope. Total tube area was quantified as the mean pixel density obtained from image analysis of five random microscopic fields using Image-J software [13].

### Statistical analyses

Data are expressed as mean ± SD. Two treatment groups were compared using Student’s *t* test. Multiple group comparisons were performed by ANOVA. GraphPad Prism version 5c for Macintosh (GraphPad Inc., La Jolla, CA, US) was used for statistical analyses. Results were considered statistically significant when *P* was < 0.05.

## Results

### Characterization of KNT cells

**Fig. 1:**
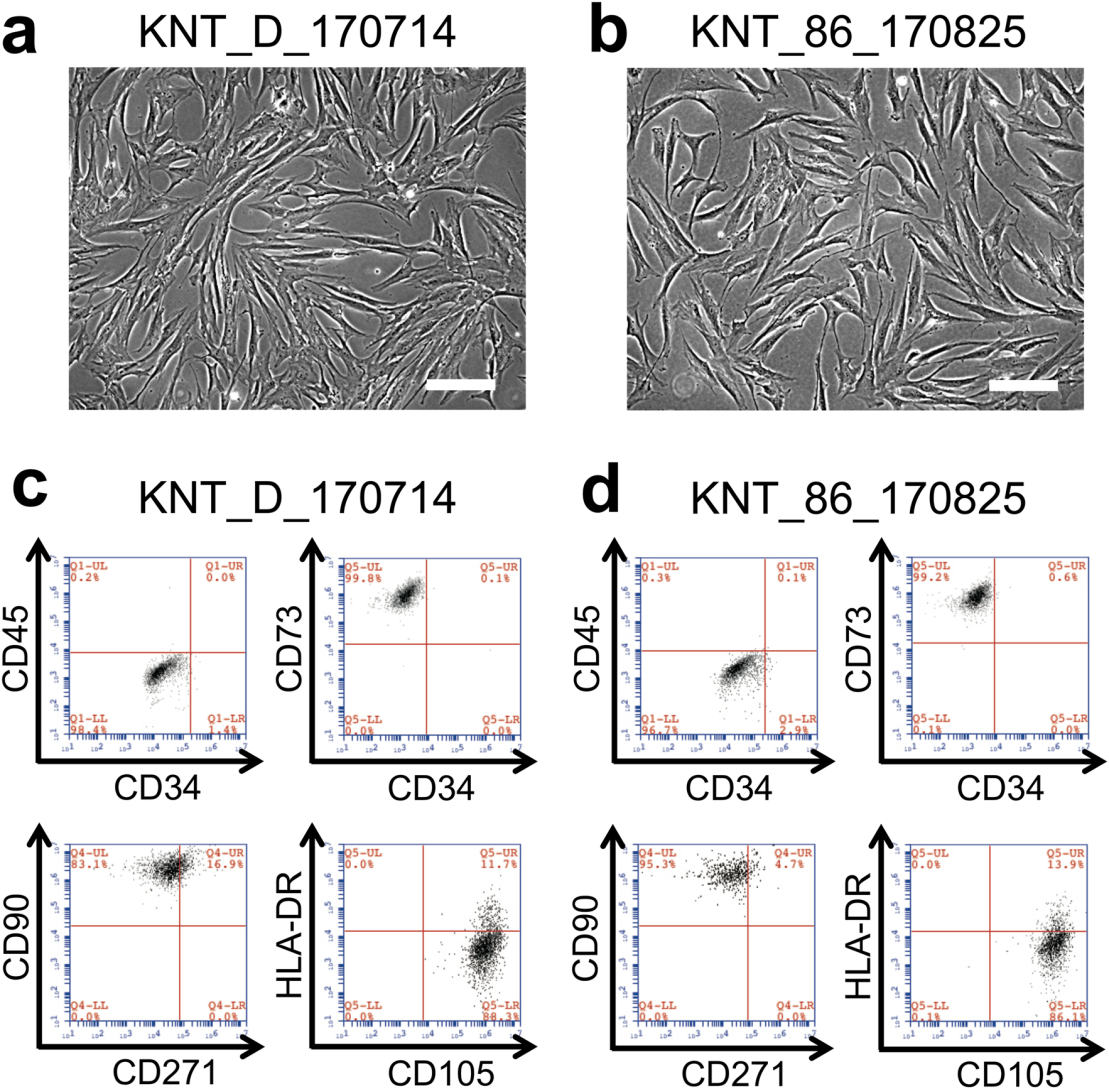
Phase contrast morphology (**a**, **b**) and cell surface markers (**c**, **d**) of BM-MSCs derived from young and elderly donors. Flow cytometric analysis of cell surface markers of KNT cells. The results confirmed that KNT cells lacked the expression of the hematopoietic markers CD34, CD45, or HLA-DR, but expressed CD90, CD73, and CD105 relative to their isotype controls (**c**, **d**). Scale bar 100 µm. Young KNT (KNT_D_176714) and elderly KNT (KNT_86_170825)

**Fig. 2:**
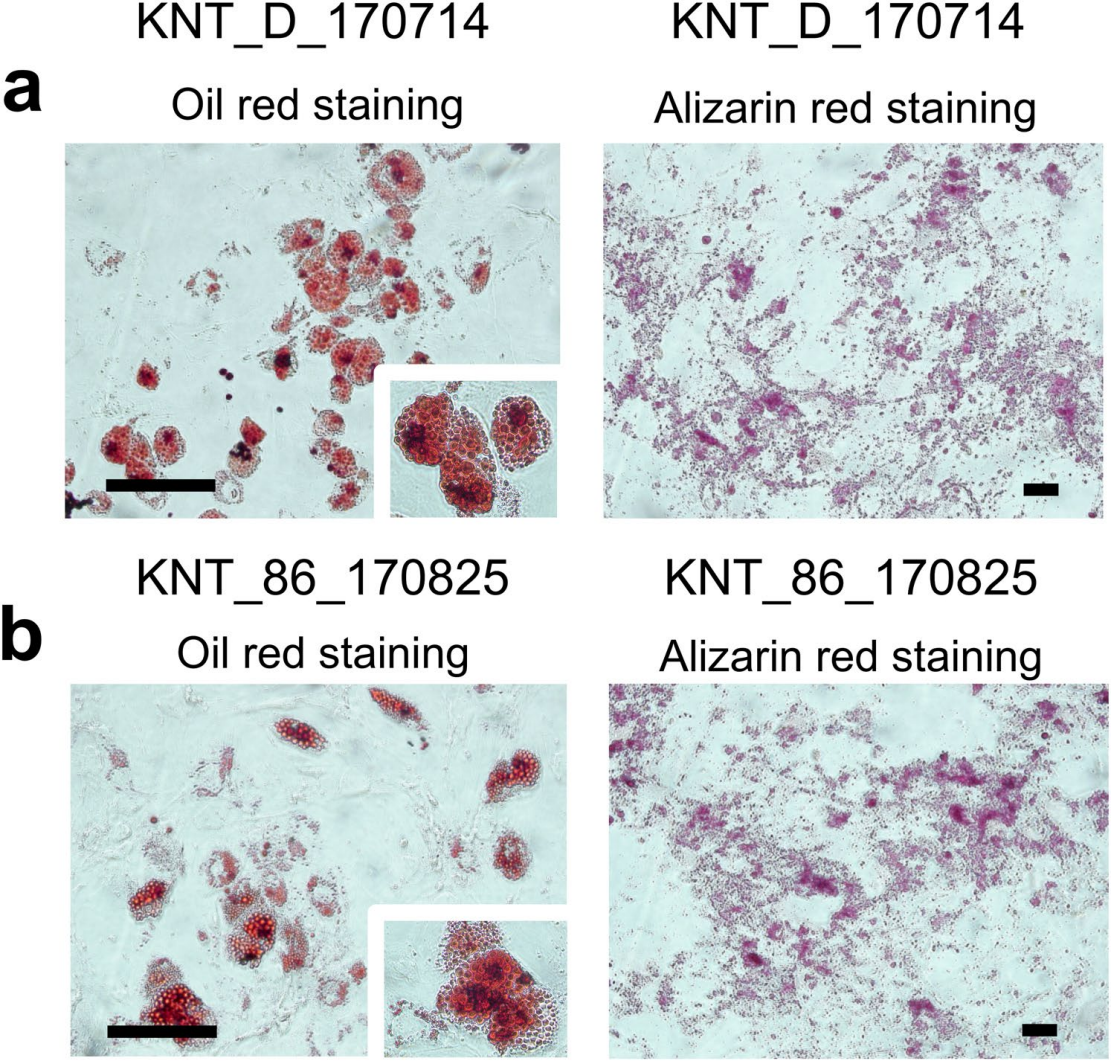
Oil red O staining reveals adipogenic differentiation, while alizarin red staining reveals osteogenic differentiation. Young KNT cells (**a**, KNT_D_170714) and elderly KNT cells (**b**, KNT_86_170825) can differentiate into adipocytes and osteoblasts. Scale bar 200 µm

KNT cells derived from young donors and those from elderly donors were microscopically compared to elucidate their morphology. KNT cells from both groups had a fibroblast-like morphology with no difference between the age groups (Fig. 1a, b). Flow cytometric analysis of the expression of CD90, CD73, and CD105, which are known as general MSC surface markers, demonstrated that KNT cells from the young and elderly groups were positive for CD90, CD73, and CD105 (Fig. 1c, d), but negative for the expression of the hematopoietic markers CD34, CD45, and HLA-DR (Fig. 1c, d). Chromosome analysis revealed normal karyotypes for both young and elderly donor-derived cells (data not shown). KNT cells from the young and elderly individuals were, respectively, analyzed for their multilineage potential using specific differentiation media. Oil Red O-stained droplets and alizarin red-stained calcium deposition were observed, confirming similar levels of differentiation to adipocytes and osteoblasts regardless of donor age (Fig. 2a, b).

### Effect of cell proliferation in the KNT cell-conditioned media (KNT cell-CM) varies among hematologic malignant cells

**Fig. 3:**
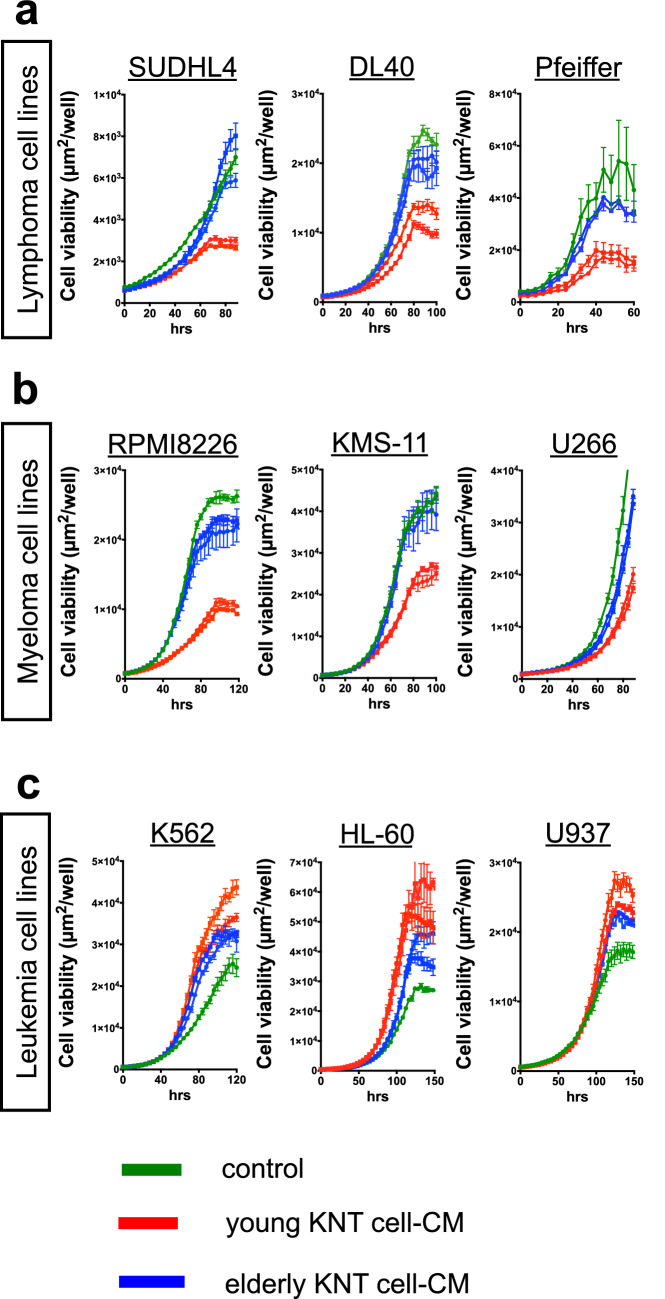
Cell viability of various hematologic neoplastic cells in response to the KNT cell-conditioned medium (CM). The green line represents the control (*n* = 3). CM from young KNT cells [red lines, (*n* = 3): KNT_D_170714 and KNT_L_170714] inhibited the growth of the lymphoma and myeloma cell lines, but enhanced the growth of the leukemia cell lines. The blue line shows the response to CM from elderly KNT cells [blue lines, (*n* = 3): KNT_80_170825 and KNT_86_170825]. Values are means ± SD of three independent experiments, with each performed on different days

We investigated the growth of cultured hematopoietic malignant cells after the addition of concentrated KNT cell-CM to elucidate their anti-neoplastic effect. In the lymphoma cell lines (SUDHL4, DL40, and Pfeiffer), the addition of young KNT cell-CM suppressed cell growth (red line, Fig. 3a) compared to the control (green line, no KNT cell-CM). Similarly, in the multiple myeloma cell lines (RPMI8226, KMS-11, and U266), adding young KNT cell-CM suppressed cell growth in a time-dependent manner (red line, Fig. 3b). In contrast, when young KNT cell-CM was added to the myeloid leukemia cell lines (K562, HL-60, and U937), cell growth was enhanced (red line, Fig. 3c). Unlike young KNT cell-CM, however, elderly KNT cell-CM did not affect tumor growth of the lymphoma and myeloma cell lines. Instead, it enhanced the growth of the myeloid leukemia cell lines (blue lines, Fig. 3a–c).

**Fig. 4:**
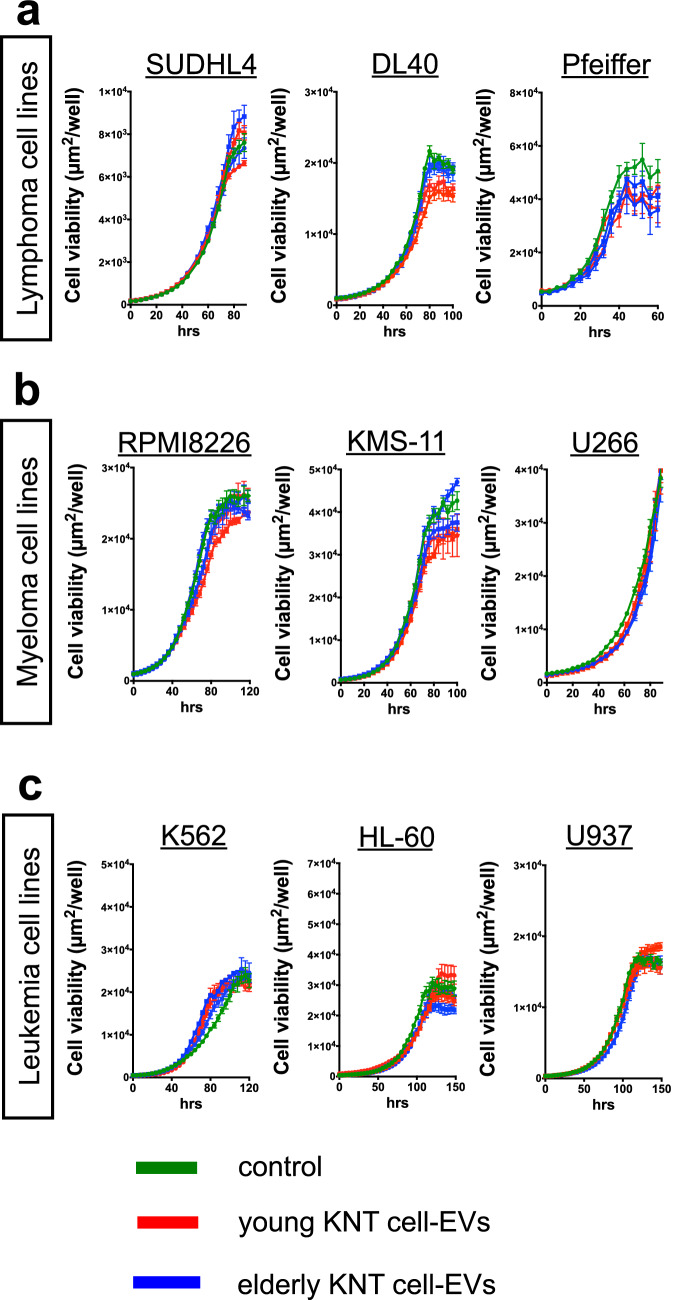
Cell viability of various hematologic neoplastic cells in response to KNT cell-extracellular vesicles (EVs). The green line represents the control. CM from young KNT cells [red lines, (*n* = 3): KNT_D_170714 and KNT_L_170714] or elderly KNT cells [blue lines, (*n* = 3): KNT_80_170825 and KNT_86_170825]. Values are means ± SD of three independent experiments, with each performed on different days

Similar to the analysis of the anti-neoplastic effects of KNT cells, hematopoietic tumor cells were cultured for 5 days after the addition of KNT cell-derived EVs. Unlike KNT cell-CM, neither the young nor the elderly donor’s KNT cell-derived EVs affected cell growth in the hematologic neoplasia cell lines (Fig. 4a–c). Therefore, CM, but not EV, from KNT cells inhibited the growth of the lymphoma and myeloma cell lines, but this effect was limited in young KNT-CM (Table 2).

**Table 2:** Summary of anti-neoplastic or angiogenic effect of KNT cells

	Conditioned medium from		Extracellular vesicles from	
	Young KNT	Elderly KNT	Young KNT	Elderly KNT
*Effect for neoplastic cell's proliferation*				
Lymphoma cells	Suppressive	Not significant	Not significant	Not significant
Myeloma cells	Suppressive	Not significant	Not significant	Not significant
Myeloid leukemia cells	Enhance	Enhance	Not significant	Not significant
*Effect for neoplasia-induced tube formation of human umbilical vessels*				
Lymphoma cells (SUDHL4)	Suppressive (*P* < 0.0001)	Suppressive (*P* = 0.0273)	Suppressive (*P* = 0.0002)	Not significant (*P* = 0.232)
Myeloma cells (RPMI8226)	Suppressive (*P* < 0.0001)	Not significant (*P* = 0.0898)	Suppressive (*P* = 0.0007)	Not significant (*P* = 0.213)
Myeloid leukemia cells (K562)	Suppressive (*P* < 0.0001)	Suppressive (*P* = 0.0044)	Suppressive (*P* = 0.0001)	Not significant (*P* = 0.387)

### Extracellular vesicles of KNT cells derived from young donors inhibit neoplasia-induced angiogenesis

**Fig. 5:**
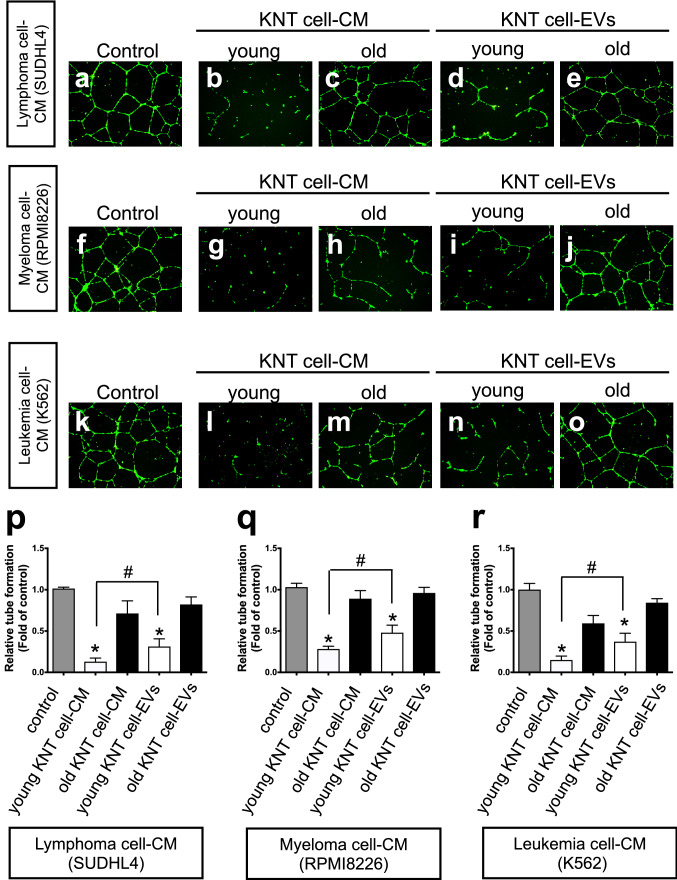
The neoplastic cell-induced tube-formation assays. Twenty-four hours after the addition of CM from KNT cells, endothelial cells were stained with calcein AM [green (*n* = 3)] and visualized under a fluorescence microscope. CM from young KNT cells inhibited angiogenesis in all cell lines. Young KNT (KNT_D_176714) and elderly KNT (KNT_86_170825). Values are means ± SD of three independent experiments, with each performed on different days

We investigated the anti-angiogenic effect using a tube-formation assay. Because of the formation of net-like vascular tubes, neoplastic cell-derived CM (control, Fig. 5a, f, k), followed by the CM or EV from KNT cells was added to evaluate angiogenesis (Matrigel + HUVEC + neoplastic cell’s CM with or without KNT-CM or KNT-EV). The addition of CM from young KNT cells, simultaneously with the start of vascular formation, profoundly inhibited tube formation compared to the controls, irrespective of the hematologic neoplastic cell type (Fig. 5b, g, p, q, r: *P* < 0.0001 vs control). Young KNT cell-derived EVs substantially suppressed neoplasia-induced tube formation (Fig. 5d, i, n), with a more evident suppression level in CM than in EV obtained from KNT cells (Fig. 5p, q, r, ^#^*P* < 0.01). The suppressive effect was not significant for KNT-EV from elderly individuals in lymphoma (SUDHL4)-induced angiogenesis (Fig. 5p, *P* = 0.232), myeloma (RPMI8226)-induced angiogenesis (Fig. 5q, *P* = 0.213), and myeloid leukemia (K562)-induced angiogenesis (Fig. 5r, *P* = 0.387) (Table 2).

## Discussion

In the current study, CM derived from KNT cells was found to affect the proliferation of hematologic neoplasia cells. In fact, CM addition suppressed the proliferation of malignant lymphoma and multiple myeloma cells, but enhanced the cell proliferation of myeloid leukemia. However, this proliferative effect on neoplastic cells was noted in CM, but not in EV. This finding indicates that the EV obtained from KNT cells, either from young or elderly donors, may not be able to affect the proliferation of hematologic neoplastic cells, which further indicates that the CM of KNT cells may contain some factors for the proliferation of neoplastic cells.

Previous studies demonstrated that MSCs differentiate into pericytes or tumor-associated fibroblasts (CAF), secreting vascular endothelial growth factor (VEGF) [14, 15], interleukin (IL)-8 [16], tumor growth factor (TGF)-β [14, 17], epidermal growth factor (EGF) [15, 18], and platelet-derived growth factor (PDGF) [14, 19], ultimately forming a tumor growth supporting microenvironment. Besides tumor progression, MSCs can suppress tumor growth through cell cycle arrest and inhibiting proliferation [20–22]. The anti-tumor properties of MSCs isolated from sources in experiments of various tumor models have been discussed [23–25]. These evidences suggest that MSCs secrete some soluble factors that influence cell growth of various types of neoplasia, with differences in proliferative potency based on target neoplastic cells.

BM-MSCs escape the rejection of heterogenous transplantation due to a deficiency in the major histocompatibility complex (MHC) [26, 27]. In addition, the EVs from BM-MSCs with a lipid bilayer display similar immunologic behavior to BM-MSCs. As EVs contain mRNA, miRNA, etc., they carry genetic elements which are transported to adjacent cells via cell-to-cell communication [28–30]. As shown here, although CM from KNT cells affects the proliferation of hematologic neoplastic cells, we cannot deny the possibility that BM-MSCs may affect the growth of minimal residual disease. Therefore, when BM-MSCs may be applied to cancer-bearing patients to control immune surveillance, the proliferative potential of neoplastic cells should be considered. Based on this study, we proposed the potential of applying BM-MSCs-derived EV to minimize the effect of neoplastic proliferation. As a result, we demonstrated that BM-MSCs derived from young donors display more potency in affecting neoplastic proliferation than those from elderly people. The age of donors for immune surveillance is, thus, a further issue that must be resolved to secure the potential of BM-MSCs for clinical application.

We assessed tube formation, representable for neoplasia-induced angiogenesis, is largely depending on donor age of KNT cells. In this study, we applied the concentrated CM derived from various hematologic neoplasias to obtain the neoplasia-induced angiogenesis control. Thereafter, we added the concentrated CM or EV from KNT cells for 24 h to evaluate their effect. Both CM and EV remarkably inhibited neoplasia-induced angiogenesis, irrespective of the types of hematologic neoplasia. Such effect of KNT cells was prominent in young-donor KNT cells, though a limited effect was notable in the CM of elderly KNT cells. Previously, we reported that a microRNA profile in EVs released by BM-MSCs is dependent on donor age [30]. We further attempted to rejuvenate EVs by introducing microRNA-340 to regain their anti-angiogenesis effect [30]. The findings indicated that the donor’s age is critical for the achievement of high therapeutic efficacy in BM-MSC cell therapy from the viewpoint of angiogenesis. In the current study, we demonstrated that young-donor-derived BM-MSC-EV and CM may be prominent anti-angiogenesis agents, irrespective of the type of hematologic neoplasia. We must consider the anti-angiogenesis effect in various types of neoplasia, including solid tumors. In addition, whether or not the EV-containing factors, such as microRNAs, are consistent among different types of neoplasia should be clarified using an enough number of BM-MSC to confirm our results. This is because the angiogenesis-targeted approach is an important issue in additional therapy for solid tumor [31, 32]. In this study, it was difficult to exclude the possibility of gender bias and obtaining enough KNT cell lines for this study proved difficult, since we could use only young males and elderly females BM-MSC due to donor’s intention.

In conclusion, we could demonstrate that the function of KNT cells is heterogenous based on donor’s age and the secreting components. Although our results did not refute the potential clinical usefulness of BM-MSC, we must consider its characteristics, especially for its use in neoplasia-bearing patients.
